# Neuregulin1/ErbB4-induced migration in ST14A striatal progenitors: calcium-dependent mechanisms and modulation by NMDA receptor activation

**DOI:** 10.1186/1471-2202-12-103

**Published:** 2011-10-12

**Authors:** Giulia Pregno, Pollyanna Zamburlin, Giovanna Gambarotta, Silvia Farcito, Valentina Licheri, Federica Fregnan, Isabelle Perroteau, Davide Lovisolo, Patrizia Bovolin

**Affiliations:** 1 Department of Animal & Human Biology, University of Turin, Via Accademia Albertina 13, Turin, Italy; 2 Neuroscience Institute of Turin (NIT), Interdepartmental Centre of Advanced Studies in Neuroscience, University of Turin, Italy; 3 Department of Anatomy, Pharmacology and Forensic Medicine and National Institute of Neuroscience-Torino, Corso Massimo D'Azeglio 52, Turin, Italy

## Abstract

**Background:**

A number of studies have separately shown that the neuregulin1 (NRG1)/ErbB4 system and NMDA-type glutamate receptors (NMDARs) are involved in several aspects of neuronal migration. In addition, intracellular calcium fluctuations play central roles in neuronal motility. Stable expression of the tyrosine kinase receptor ErbB4 promotes migratory activity in the neural progenitor cell line ST14A upon NRG1 stimulation. In this work we analyzed the potential interactions between the NRG1/ErbB4 system and NMDARs in the ST14A migratory process as well as its calcium dependence.

**Results:**

RT-PCR studies have shown that both native ST14A cells (non-expressing ErbB4), as well as ErbB4-transfected cells express low levels of a restricted number of NMDAR subunits: NR1, NR2C, NR2D and NR3B. The resulting NMDAR would form Ca^2+ ^channels characterized by low Mg^2+^-sensitivity and low Ca^2+^-permeability, generating small, long-lasting currents. Ca^2+^-imaging experiments showed slow [Ca^2+^]_i _increases in 45% of the cells following 8 μM NMDA stimulation. Basal migration of ErbB4-transfected ST14A cells was unaffected by 18 hrs NMDA incubation. However, over the same incubation time, NMDA was able to significantly enhance NRG1-induced migration. Pre-incubation with the intracellular calcium chelator BAPTA-AM reduced both NRG1- and NRG1/NMDA-stimulated migration, suggesting the involvement of Ca^2+ ^in these processes. NRG1 stimulation of ErbB4-transfected ST14A cells induced a sustained, long-lasting increase in [Ca^2+^]_i_, in 99% of the cells. These intracellular Ca^2+ ^signals could be ascribed to both release from intracellular stores and influx from the extracellular medium trough a mechanism of store-operated calcium entry (SOCE). Short-time co-incubation of NMDA and NRG1 did not substantially modify the NRG1-induced intracellular calcium signals.

**Conclusions:**

In summary, NRG1 stimulation of the ErbB4 receptor exerts a sustained [Ca^2+^]_i _increase in ST14A neural progenitors; NRG1-induced migration is Ca^2+^-dependent and can be positively modulated by activation of the NMDA receptor.

## Background

Neuroblast migration is a complex, integrated process that involves a coordinated sequence of cellular events, from surface receptor activation by external stimuli to transduction of these stimuli by intracellular pathways and subsequent dynamic cytoskeleton remodelling [1]. Many signalling molecules participate in the modulation of neuronal migration, including molecules classified as neurotransmitters and as growth/differentiation factors [2,3].

The tyrosine kinase receptor ErbB4 binds members of the Neuregulin (NRG) family of trophic and differentiation factors. Receptor-ligand interaction induces the homo- or heterodimerization with other members of the ErbB receptor family, which in turn results in the activation of several intracellular signalling pathways and the induction of cellular responses including migration of neuronal precursor cells [4-6]. *In vivo *studies demonstrate that the NRG1/ErbB4 system is involved in tangential migration of olfactory bulb (OB) interneuron precursors in the rostral migratory stream (RMS) [7] and of cortical interneuron precursors migrating from the ventral telencephalon [8].

The N-methyl-d-aspartate (NMDA)-type glutamate receptor (NMDAR) is a calcium permeable channel whose functions have been mainly characterized in excitatory synapses of mature neurons. Recent studies strongly suggest that glutamate may act as a paracrine signal through NMDARs early expressed by neuronal precursors and immature neurons [9]. NMDARs also appear to be involved in several modes of migration *in vivo*, such as radial migration of cerebellar granule cells and tangential/radial migration in the developing cortex [10-14].

In the last twenty years, several studies have pointed to a key role of Ca^2+ ^signalling in the control of neuronal migration. Evidence came first from imaging studies of granule cell migration in acute mouse cerebellar slices, in which blockade or enhancement of Ca^2+ ^influx through N-type Voltage Dependent Calcium Channels (VDCCs) or the NMDAR reduced or promoted the rate of granule cell movement, respectively [10,15]. Similarly, the involvement of calcium influx from the extracellular medium has also been reported for the migration of other cell types like GNRH-1 neurons, cortical projection neurons and interneurons [13,16-18].

In developing and mature neurons, NMDARs and the NRG1/ErbB4 system have been found to interact in several ways. In the cerebellar cortex, NRG1 release by afferent fibres induces the expression of NR2C subunits during synaptogenesis through the activation of ErbB4 receptors [19]; in the rat prefrontal cortex and in neuronal primary cultures, NRG1 activation of ErbB4 modulates NMDAR currents and regulates trafficking and endocytosis of NMDAR subunits at excitatory synapses [20]; NRG1 signalling through Fyn kinase modulates NMDAR phosphorylation [21]; activity-driven glutamatergic synapse development is impaired by genetic deficits in NRG1/ErbB4 signalling [22].

Up to now, no studies have analyzed the interaction between these two systems in neuronal migration. The present work is a first attempt to address this issue in a simplified *in vitro *model of neural progenitors, the ST14A cell line [23], in which NRG1/ErbB4 mediated migration has been previously characterized [6,24]. As expected from the expression data, we show that NMDA induces small Ca^2+ ^signals in ErbB4-transfected ST14A cells. While NMDA does not modify basal migration rates, it is able to increase NRG1-induced migration. We provide evidence that both NRG1- and NRG1+NMDA-induced migrations are calcium-dependent processes. Indeed, NRG1 stimulation of ErbB4 receptors elicits long lasting increases in [Ca^2+^]_i_, with a mechanism involving store-operated calcium entry (SOCE). Short-time (minutes) co-incubation of NMDA and NRG1 is not sufficient to significantly modify the NRG1-induced calcium signals, suggesting a complex mechanism of interaction requiring either a longer time span or the generation of calcium signals confined to subcellular domains not detectable with recordings of changes in bulk cytosolic calcium concentration.

## Results

### NMDA does not modify basal migration of wild type ST14A cells

#### NMDAR expression in wild type (wt) ST14A cells

**Figure 1:**
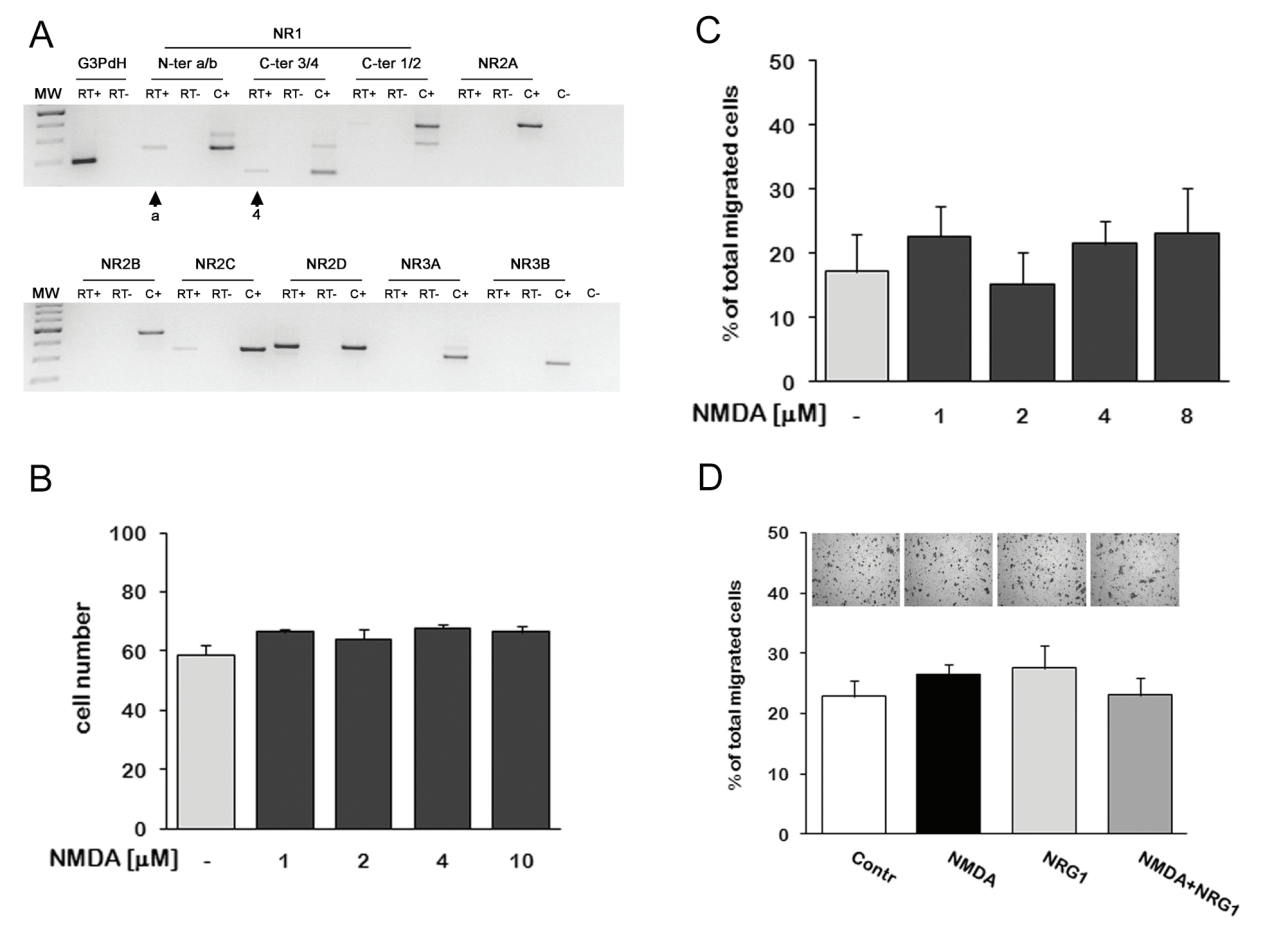
**NMDA and NRG1 do not modify basal migration of wild-type ST14A neural progenitors**. **A:** Agarose gel electrophoresis of RT-PCR amplification products. Wt ST14A cells were cultured in 2% FBS DMEM for 18 hrs before total RNA isolation. Splicing-specific primer pairs were employed to recognize N-terminal (1.a/1.b) and C-terminal (1.1/1.2; 1.3/1.4) splicing sites of the NR1 subunit. Wt ST14A express NR1.4a (combination of 1.a N-term and 1.4 C-term splicing forms, see arrows), NR2C and NR2D subunits. No PCR product was observed in the absence of reverse transcriptase (RT-) or cDNA (C-, water). "C-" reactions contained all primer pairs. C+ = various brain regions from adult rats. **B:** NMDA dose-response assay on wt ST14A cells. Cells were incubated in increasing concentrations of NMDA (1, 2, 4, 10 μM) in 2% FBS DMEM for 18 hrs. Data are expressed as mean + standard deviation (SD, n = 3). **C: **Effect of NMDA on wt ST14A cell migration measured by transwell assays performed in 2% FBS DMEM for 18 hrs with different concentrations of NMDA. **D:** wt ST14A migration was measured by transwell assay with addition of NMDA (8 μM) and NRG1 (5 nM) either alone or in combination. Representative images of migrated cells are shown above each bar/treatment. Migration was calculated as percentage of total migrated cells (see Material and Methods). Biological triplicate experiments were carried out in technical triplicate. Data are presented as means + SD. No statistically significant difference was found among different treatments.

Data from several groups have suggested that neural stem cells and neural progenitors may express low levels of the NMDA receptor [25,26]. We tested the expression of all known NMDAR subunits in wt ST14A cells by RT-PCR. wt ST14A cells were grown at 33°C for 18 hrs in 2% FBS DMEM (same culture conditions as those employed for subsequent migration assays) and were harvested for RNA extraction. Figure 1A shows that ST14A cells express the mRNAs of a restricted number of NMDAR subunits: NR1, NR2C and NR2D. The use of splicing-specific NR1 primer pairs allowed us to determine that the most expressed isoform is NR1.4a. This particular NR1 isoform lacks the N1 cassette (coded by exon 5) and has a long intracellular tail lacking the ER retention signal and containing a PDZ interacting motif.

#### NMDA dose-response assay on viability of wt ST14A cells

Before testing the effects of NMDAR stimulation on wt ST14A progenitor migration, we checked whether NMDA could modify the total number of cells at the concentrations employed in the subsequent migration assays. NMDA at high concentrations can be cytotoxic, depending on receptor density and subunit composition [27]. NMDA could also increase cell proliferation and/or survival, as demonstrated by several groups by *in vivo *and *in vitro *experiments [28,29].

Wt ST14A cells were incubated with scalar concentrations (1-10 μM) of NMDA for 18 hrs (the time length used in all migration assays), then fixed and counted. Results show that NMDA does not modify the total number of cells (Figure 1B). This result suggests that, in the concentration range 1-10 μM, NMDA has no excitotoxic effects on wt ST14A cells. We consider very unlikely the occurrence of proliferative effects, due to the low-serum culture conditions (2% instead of standard 10% serum) and the short incubation time (18 hrs) used in these experiments. Compensatory effects of concurrent cell division and cell death are also very unlikely; therefore, it can be safely assumed that the 1-10 μM NMDA concentration range is suitable for migration assays.

#### Transwell assays on NMDA-stimulated wt ST14A cells

ST14A cells have been generated from E14 striatum primordia. Therefore, we can assume that they mostly derive from the lateral ganglionic eminence, a source of a large number of migrating neuronal precursors. Wt ST14A cell motility in un-stimulated conditions has been previously described by other laboratories [30].

On this basis, we tested the hypothesis that NMDA can modulate basal migration of wt ST14A cells, either positively or negatively. Cells were treated for 18 hrs with scalar concentrations of NMDA in a transwell assay. Results clearly show that wt ST14A cells have a low basal intrinsic motility that is not altered by the presence of NMDA (Figure 1C). On the other hand, NMDA could have no effect per se in ST14A cell migration, and have instead an effect when added together with other molecules, such as members of the neuregulin family, and specifically NRG1, that has been shown to promote ErbB4 mediated neuronal migration [6-8]. Wt ST14A cells do not express ErbB4; however, they express other ErbB receptors, which could mediate a NRG1-induced response in the presence of NMDA.

To test these hypotheses, we performed transwell assays stimulating cells either with NRG1 alone or in combination with 8 μM NMDA (Figure 1D). As expected [6], NRG1 does not enhance migration of wt ST14A, both when administered alone or in the presence of NMDA.

### NMDA enhances NRG1-induced migration in ErbB4 transfected-ST14A cells

Our laboratory has previously shown that ST14A clones stably transfected with different ErbB4 isoforms show a variable degree of increased migratory activity when stimulated by NRG1, depending on the specific ErbB4 isoform [6]. Since there are no data in the literature comparing the interactions of different ErbB4 isoforms with NMDARs, we used the two ErbB4 expressing clones showing the highest increase in migration following NRG1 stimulation. These clones express the JMa/cyt2 ErbB4 isoform, containing a cleavage site for the metalloprotease TACE in the juxtamembrane region, and the JMb/cyt1 ErbB4 isoform, that has an intracellular binding site for PI3K. The latter, however, showed a very high basal migratory activity in preliminary experiments, making it more difficult to unmask the effect of any molecule added to NRG1. Therefore, we chose a JMa/cyt2 ErbB4 expressing clone (the clone A2.1, from now on called "ErbB4-transfected cells") characterized by a lower level of basal migration.

**Figure 2:**
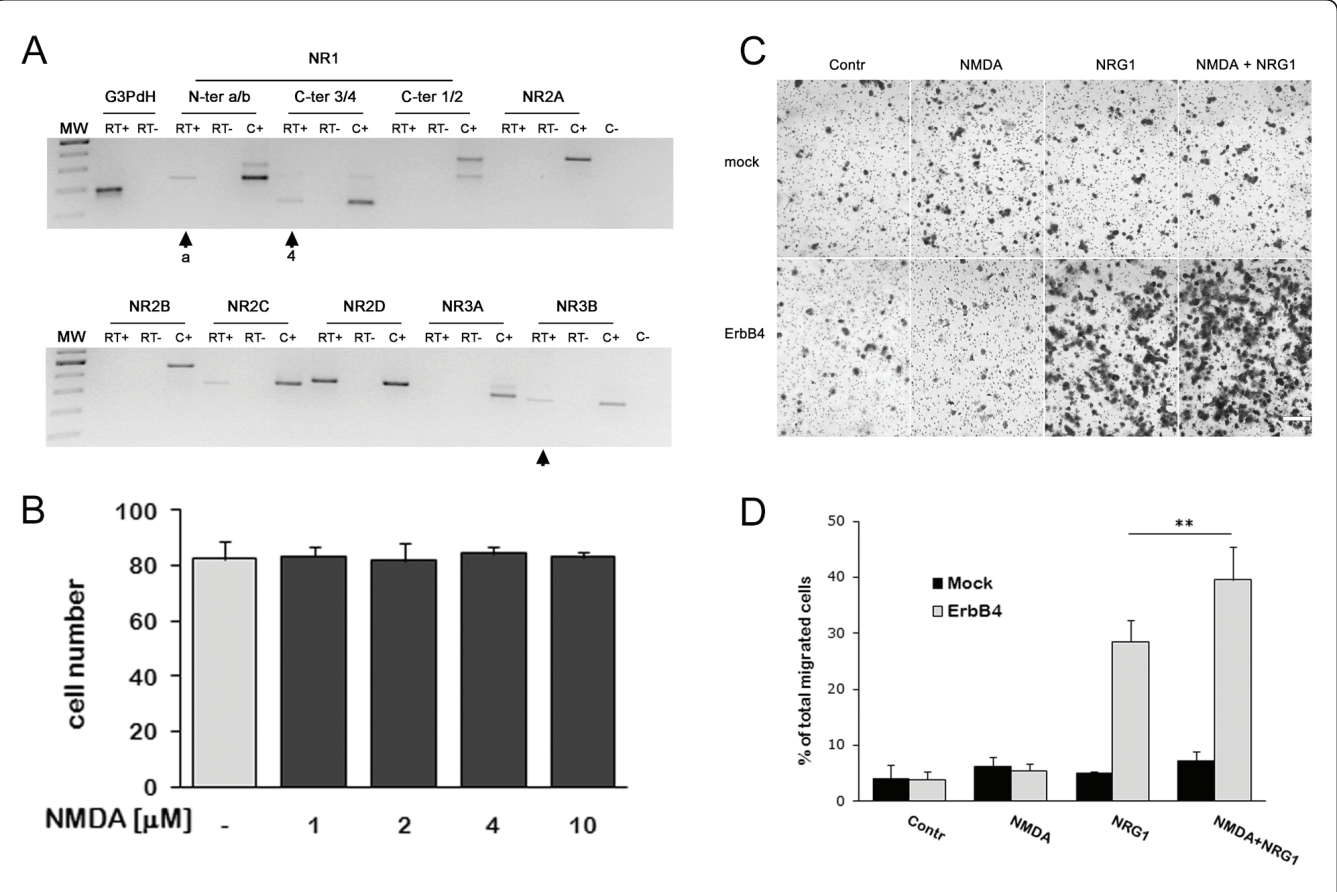
**NMDAR activation modulates NRG1-induced migration in ErbB4-transfected ST14A cells**. **A: **ErbB4-transfected cells were cultured in 2% FBS DMEM for 18 hrs before total RNA isolation. Agarose gel electrophoresis was used to separate RT-PCR amplification products obtained with primer pairs specific for different NMDAR subunits (see Figure 1 for details). ErbB4-transfected cells express NR1.4a (arrows), NR2C, NR2D and NR3B (arrow). Note that NR3B was absent in wt ST14A cells. No PCR product was observed in reactions that omitted either reverse transcriptase (RT-) or starting material (C-, water). C- reaction contained all primer pairs. C+ = various brain regions from adult rats used as positive controls. **B: **As for wt ST14A cells, NMDA (1-10 μM) did not change ErbB4-transfected cell number. Cells were incubated in increasing concentrations of NMDA in 2% FBS DMEM for 18 hrs. Data are expressed as mean + SD (n = 3). **C: **Representative images of migrated cells showing that NMDA enhances NRG1-induced migration in ErbB4-transfected cells. Cells were treated either with 8 μM NMDA, 5 nM NRG1 or both for 18 hrs. Scale bar represents 200 μm. **D: **Histogram of the transwell experiment shown in C. Migration was calculated as percentage of total migrated cells as described in Material and Methods. Biological triplicate experiments were carried out in technical triplicate. Data are presented as means + SD. Statistical analysis was performed by applying two-way ANOVA, followed by Tukey's posthoc test (see text for details), ** = p < 0.01.

As we did for wt ST14A cells, we tested the effect of scalar concentrations of NMDA on the total number of cells for 18 hrs. Again, we found that NMDA, in the concentration range 1-10 μM, does not modify the total number of cells (Figure 2B).

We subsequently performed transwell assays on ErbB4-transfected cells stimulated with 8 μM NMDA, or 5 nM NRG1, or the two drugs in combination, using mock cells as a negative control (Figure 2C-D). Statistical analysis with two-way ANOVA revealed significant main effects of drug treatment (F = 64.165, df = 3,16, P = 3.88E-09) and of ErbB4 expression (F = 138.639, df = 1,16, P = 2.71E-09) and significant interaction between the 2 factors (F = 50.474, df = 3,16, P = 2.22E-08). Posthoc comparison (Tukey's test) showed that mock cells do not show significant changes as compared to control when stimulated with NRG1 alone, NMDA alone, or the combination NRG1+NMDA; these results are in line with those obtained with wt ST14A cells (Figure 1D). In ErbB4-transfected cells, as expected, NRG1 induces a highly significant increment in the number of migrated cells (P = 2.24E-07), while NMDA alone does not significantly alter basal migration (P = 0.986). Intriguingly, the combination of NMDA and NRG1 results in a significantly higher migration rate as compared to that observed with NRG1 alone (38% increase, P = 0.0056). The addition of the NR2C/D subunit-specific antagonists PPDA (0.5 μM) and UBP141 (5 μM) was able to decrease the combined effects of NRG1 and NMDA by a similar extent (35-40% decrease of NRG1+NMDA stimulated migration; see Additional files 1 and 2: Figure S1 and S2). The channel blocker MK-801 (10 μM) and the competitive antagonist D-AP5 (30 μM, acting at the glutamate binding site on all NR2 subunits with affinity ranking: NR2A≈2B > 2C≈2D) were also able to reduce migration, albeit to a lesser extent (Additional file 1: Figure S1). Potential effects of all 4 NMDA antagonists on cell number and viability were previously checked in a dose-response assay over an 18 hrs period (data not shown), to make sure that the agonist concentration used in migration assays would not affect cell survival and cell proliferation.

To better characterize the NMDAR subunits expressed by ErbB4-transfected ST14A cells and compare them to wt cells, we performed RT-PCR studies with NMDAR subunit specific primers. Results indicate that, as for wt ST14A cells, the ErbB4-transfected clone expresses NR1.4a, NR2C and NR2D transcripts (Figure 2A). In addition, the NR3B subunit mRNA was found. Generally, NMDARs are composed of at least one NR1 subunit and variable combinations of NR2/NR3 subunits, generating NMDA receptors differing in their functional characteristics and in cell-type and sub-cellular localization [31]. The two NR2 subunits expressed by ST14A cells (NR2C and NR2D) are prevalently extra-synaptic and are characterized by low-conductance openings and low sensitivity to Mg^2+ ^block. NR3 subunits act like a dominant negative, reducing the amplitude of the response and its desensitization, and decreasing the Ca^2+ ^permeability of the NMDAR channel. We also quantified the expression of NMDAR subunit mRNAs in mock and ErbB4-transfected ST14A cells stimulated with NRG1, NMDA and the two molecules together (data not shown); we found no evidence of regulation of NR1, NR2C, NR2D and NR3B subunit expression. Whether the NMDAR is regulated at a different level (subunit translation, assembly, localization, phosphorylation) remains to be elucidated in future studies.

In summary, migration experiments performed with NMDAR subunit-specific antagonists and expression studies together suggest that NR1/NR2C/D-containing NMDARs synergistically interact with the NRG1/ErbB4 system, ultimately producing a potentiation of ST14A cell migration. A possible functional role of the NR3B subunit remains to be ascertained.

### Calcium is involved in NRG1-induced migration

#### *BAPTA-AM dose-response assay on viability *of ST14A cells

Neurotrophins, neurotransmitters and other extracellular signalling molecules have been shown to promote neuronal motility in a calcium-dependent way [12,32]; for this reason, we checked whether the migratory activity induced by NRG1, and its further enhancement observed when NMDA is added to NRG1, are Ca^2+^-dependent.

To evaluate the role of Ca^2+ ^we treated cells with BAPTA-AM, a Ca^2+ ^chelator that can cross the membrane and, once in the cytoplasm, is cleaved by endogenous esterases thus buffering the increases in [Ca^2+^]_i _due to extracellular influx or intracellular release from stores. Just because of its pleiotropic effect, buffering intracellular Ca^2+ ^can be dangerous and can affect processes like cell adhesion to substrata or cell survival. To test the effect of BAPTA-AM on cells, ErbB4-transfected cells were incubated with scalar concentrations of BAPTA-AM (5, 10, 15 μM) and the number of cells after 18 and 24 hrs was evaluated (data not shown). Since even during the 24 hrs incubation the cell number was not affected by the application of BAPTA-AM and no signs of cell detachment were evident, we chose the higher concentration (15 μM) for the subsequent transwell assays.

#### Transwell assays on NMDA/NRG1-stimulated ErbB4-transfected cells in the presence of BAPTA

**Figure 3:**
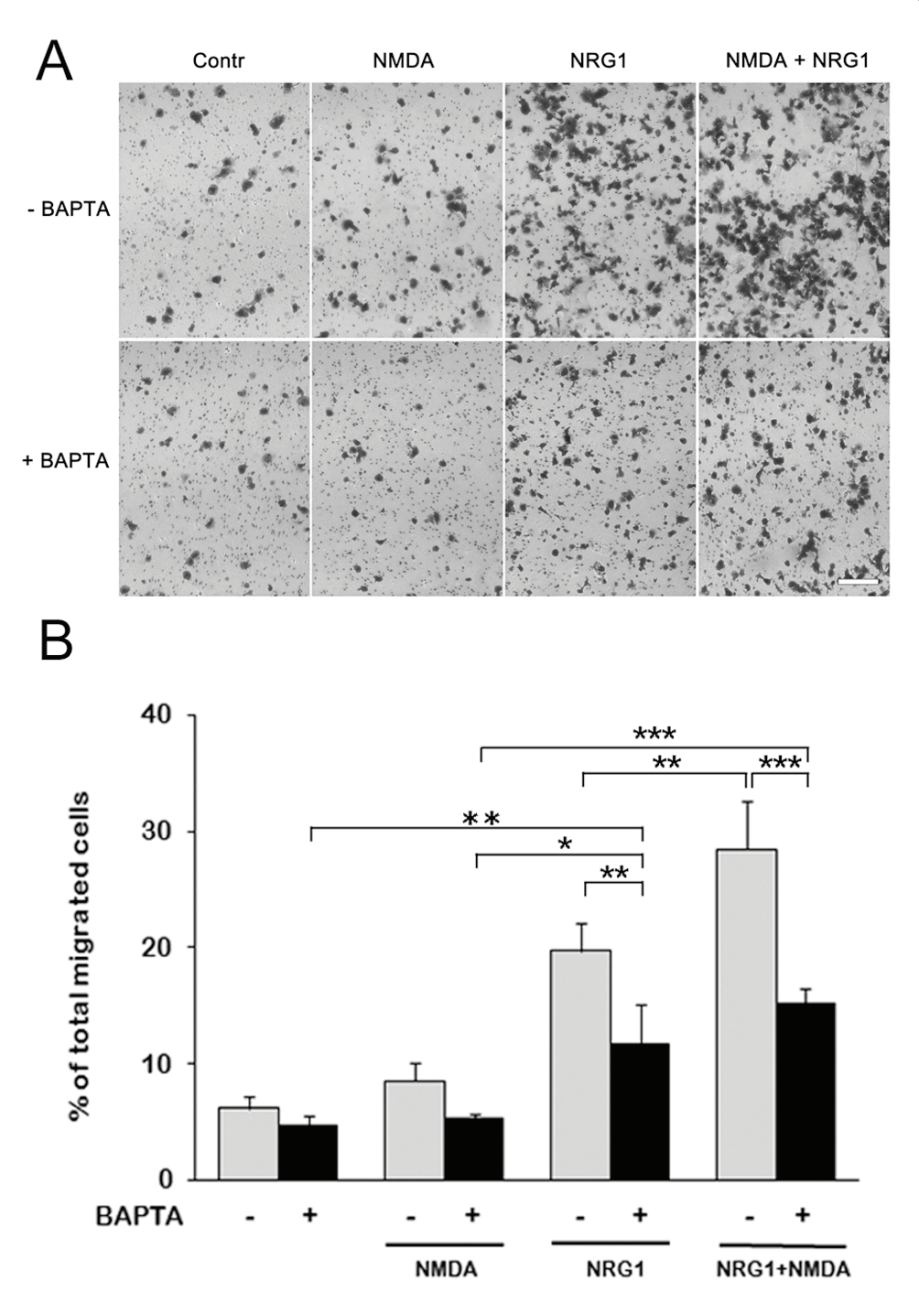
**Calcium is involved in NRG1-induced migration**. **A: **Representative images of migrated ErbB4-transfected cells in the presence/absence of 15 μM BAPTA-AM, showing that both NRG1- and (NRG1+NMDA)-induced migrations are reduced by buffering of intracellular Ca^2+^. Cells were treated for 18 hrs with 8 μM NMDA, 5 nM NRG1 or both. **B:** Histogram of the same migration assay. Migration was calculated as percentage of total migrated cells as described in Material and Methods. Biological triplicate experiments were carried out in technical triplicate. Data are presented as means + SD. Statistical analysis was performed by applying two-way ANOVA, followed by Tukey's posthoc test (see text for details) * = p < 0.05; ** = p < 0.01; *** = p < 0.001.

Transwell assays were performed on ErbB4-transfected cells previously incubated with 15 μM BAPTA-AM in 2% FBS-DMEM for 20 minutes (Figure 3A). Statistical analysis with two-way ANOVA showed significant main effects of drug treatment (F = 78.713, df = 3,16, P = 8.53E-10) and of intracellular Ca^2+ ^chelation (+/-BAPTA, F = 54.394, df = 1,16, P = 1,57E-06), with significant interaction between the two factors (F = 8.939, df = 3,16, P = 0.0010). Posthoc comparison (Tukey's test) showed that BAPTA-AM had no effect on migration either in control conditions (P = 0.993) or in the presence of NMDA (P = 0.560). Interestingly, BAPTA-AM significantly decreased NRG1-induced migration (40% reduction, P = 0.006) without abolishing it completely (Figure 3B), implying that this migratory mechanism is at least in part Ca^2+^-dependent. Furthermore, in the presence of BAPTA-AM, the NRG1 and NRG1+NMDA conditions were not significantly different (P = 0.450), meaning that BAPTA-AM is able to prevent the cooperation between the pathways activated by NMDA and NRG1 (Figure 3B).

The addition of the NR2C/D subunit-specific antagonists UBP141 (5 μM) did not further potentiate the inhibitory effect of BAPTA (Additional file 1, Figure S2), suggesting that this intracellular chelator is able to buffer the Ca^2+ ^entering the cell through the NMDAR.

Collectively, these data suggest that intracellular BAPTA-AM, by buffering [Ca^2+^]_i _changes, interferes with the NRG1-induced migration and antagonizes the synergistic effect exerted by NMDA on NRG1.

#### Calcium signals induced by NRG1 and NMDA

**Figure 4:**
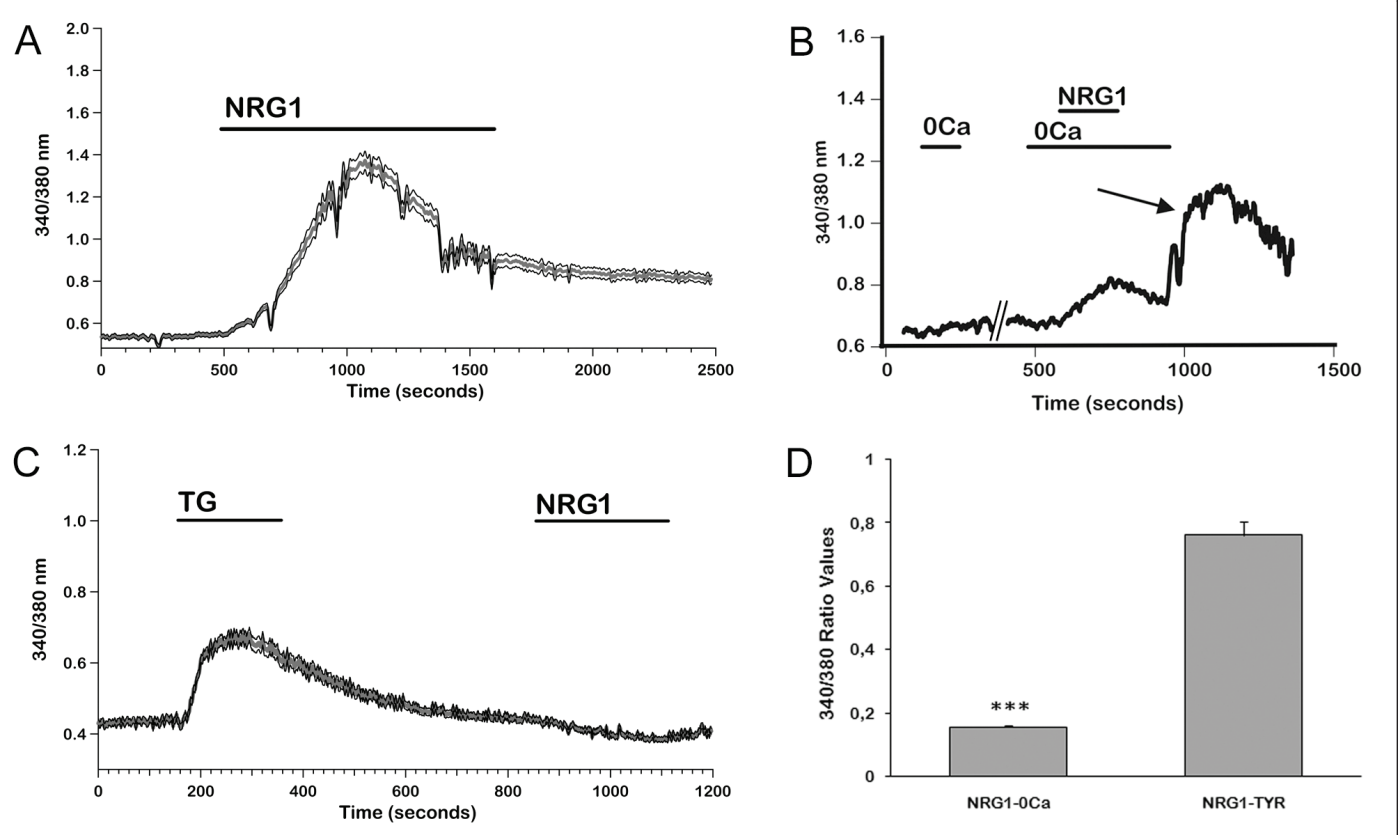
**Acute stimulation with 5 nM NRG1 induces [Ca^2+^]_i _increases in ErbB4-transfected cells**. ErbB4-transfected cells were loaded with 3 μM FURA-2-AM and perfused with a Tyrode Standard solution with or without calcium in the presence of 5 nM NRG1 in order to characterize the [Ca^2+^]_i _changes induced by the factor. **A:** Mean ± SE from a representative experiment (n = 75), showing calcium induced by 5 nM NRG1 in the presence of 2 mM extracellular [Ca^2+^]. **B:** Example of a response to 5 nM NRG1 in the absence of extracellular Ca^2+ ^(0Ca). NRG1 induced a slow increase due to release from intracellular stores; reintroduction of 2 mM [Ca^2+^] in the external bath induced a sustained increase of [Ca^2+^]_i _(arrow). **C:** Lack of response to NRG1 in cells in which intracellular stores had been depleted by means of 0.1 mM thapsigargin (TG). Mean ± SE from a representative experiment (n = 19). **D:** Histogram representing mean values of maximum amplitude (ΔR) responses to NRG1 in Tyrode Standard solution and in the same solution without Ca^2+ ^(n = 164 in Tyrode solution without Ca^2+ ^; 119 in Tyrode Standard). Data represent means + SE from three independent experiments. *** p < 0.001 vs NRG1-Tyr.

Subsequently, we investigated if NRG1 stimulation in ErbB4-transfected cells could elicit calcium signals. As shown in Figure 4A, 5 nM NRG1 caused an elevation in intracellular calcium. Responding cells were 99% (n = 454); mean peak amplitude was 0.68 ± 0.03 (Figure 4D). In most cells, the signal was biphasic, a transient phase being followed by a long lasting plateau. When the agonist was washed out, [Ca^2+^]_i _remained at levels higher than control for tens of minutes in most cells (Figure 4A); in some cells, it returned slowly to basal levels.

In order to distinguish the relative contributions of extracellular Ca^2+ ^influx and release from intracellular stores, we performed the NRG1 stimulation in an extracellular solution without Ca^2+ ^and with 0.5 mM EGTA added. Figure 4B shows that in the absence of extracellular Ca^2+ ^a lower and slowly decaying signal can be observed, implying a contribution of Ca^2+ ^released from intracellular stores (responding cells 94%, n = 170; peak amplitude 0.16 ± 0.01; see Figure 4D).

When Ca^2+ ^was restored into the perfusion solution, after NRG1 had been removed from the bath, a strong increase in [Ca^2+^]_i _was observed in 78% of cells (Figure 4B, arrow). Mean ΔR was 0,34 ± 0,01. This effect can be ascribed to store-operated calcium entry/SOCE [33], *i.e*. to the influx of extracellular Ca^2+ ^triggered by depletion of intracellular stores.

This mechanism may account, either completely or partially, for the long lasting plateau observed in 2 mM extracellular Ca^2+^. To clarify this issue, we stimulated the cells with 0.1 mM thapsigargin (TG) and, subsequently, during the plateau phase of the response, with 5 nM NRG1. TG induces irreversible depletion of ER calcium stores and activates SOCE. With this protocol, NRG1 did not elicit any additional increase in [Ca^2+^]_i _in 77 out of 78 cells responding to TG with a biphasic response (transient followed by plateau), providing evidence that the influx activated by NRG1 is almost totally dependent on SOCE (Figure 4C).

When NRG1 was applied to mock transfected cells in the presence of 2 mM extracellular Ca^2+^, no response could be observed (n = 304; data not shown).

**Figure 5:**
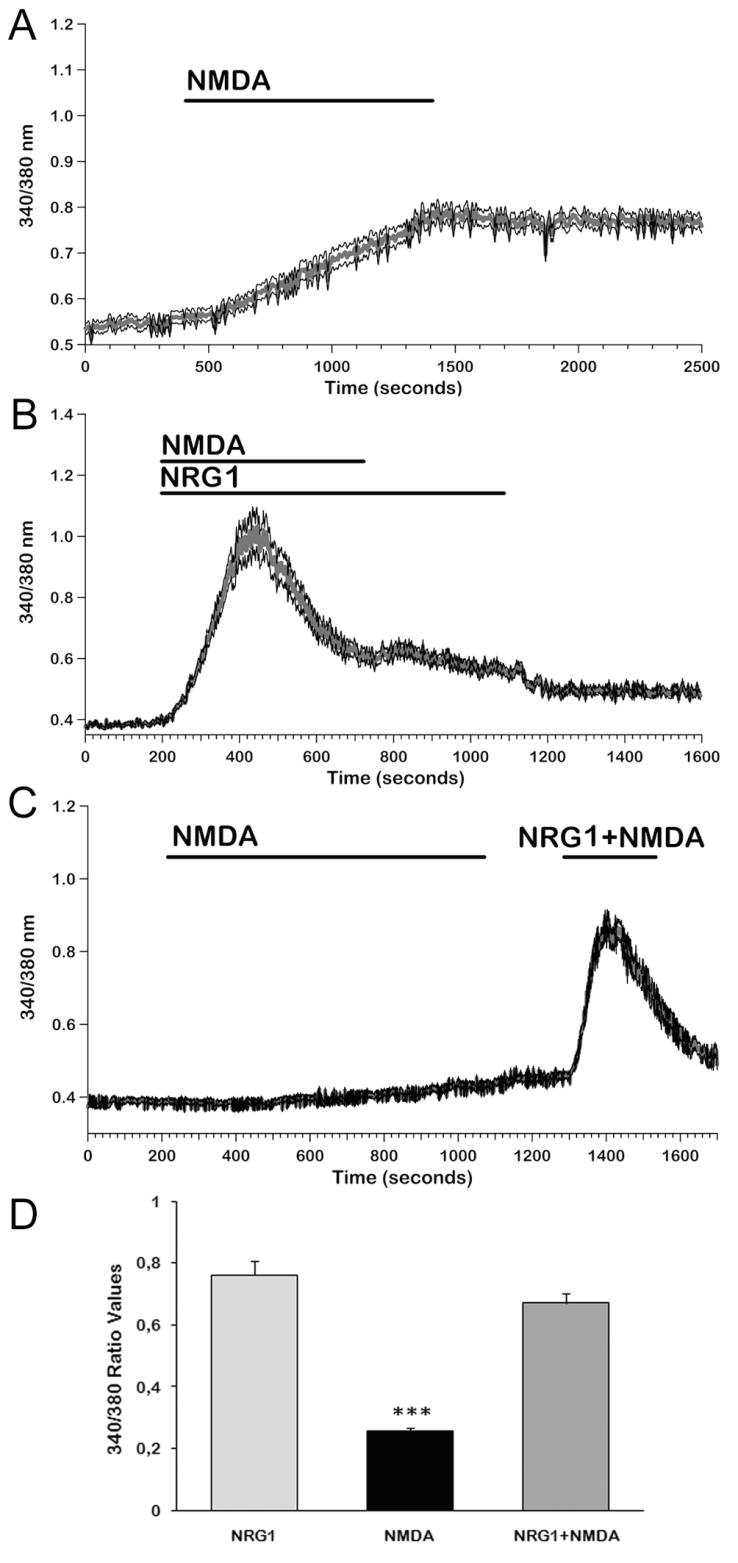
**[Ca^2+^]_i _responses of ErbB4-transfected cells to NMDA and NMDA+NRG1**. We measured [Ca^2+^]_i _signals induced after an acute stimulation with 8 μM NMDA and we compared them with the ones obtained co-stimulating ErbB4-transfected cells with NMDA and NRG1. **A:** Increases in [Ca^2+^]_i _induced by 8 μM NMDA in the presence of 2 mM extracellular [Ca^2+^] differ from those elicited when NRG1 is perfused: NMDA induced a very slow rise in [Ca^2+^]_i _and the plateau was reached after several minutes. Mean ± SE from a representative experiment (n = 69). **B:** The increase in [Ca^2+^]_i _induced by co-stimulation with NRG1 and NMDA showed the same amplitude and time course as NRG1-induced signals. Removal of NMDA slightly affected the plateau phase. Mean ± SE from a representative experiment (n = 82). **C:** When NRG1 was added during the slow rising response to NMDA, a standard biphasic increase in [Ca^2+^]_i _could be observed showing the same mean amplitude as with NRG1 alone. Mean ± SE from a representative experiment (n = 90). **D: **Histogram representing mean values of maximum amplitude (ΔR) responses to NMDA, NRG1 and NMDA+NRG1 in Tyrode Standard solution. (n = 197 with NRG; 118 with NMDA and 216 with NMDA+NRG1). Data represent means + SE from three independent experiments. *** p < 0.001 vs NRG1 and NRG1+NMDA.

On the other hand, stimulation with 8 μM NMDA led to a very slow increase in [Ca^2+^]_i _in 46% of cells tested (n = 247, Figure 5A), reaching a value of 0.26 ± 0.01 (Figure 5D). More than 10 min were needed to reach the plateau. This result is coherent with the low NMDAR expression level found in the ST14A cells, and the peculiar time course of the response can be explained by the reduced Mg^2+ ^dependence of the receptors present in these cells, that can allow for the opening of some channels even at resting membrane potentials; this activation may induce a slow depolarizing response, that in turn may cause further channel openings.

The specificity of the NMDA-induced [Ca^2+^]_i _elevation was checked by employing the NR2C/D subunit-specific antagonist UBP141. Due to the slow time course and limited reversibility of the responses to NMDA, we did not test UBP141 in acute protocols. Rather, we preincubated the cells with the blocker (10 μM); following stimulation with 8 μM NMDA, no response could be detected (n = 57 cells, see Additional file 3: Figure S3).

To test the dependence of the observed increases in [Ca^2+^]_i _from calcium influx from the extracellular medium, cells were preincubated in a calcium-free extracellular solution and subsequently challenged with 8 μM NMDA: no response could be observed (n = 80 cells, see Additional file 4: Figure S4).

We subsequently studied the effects of co-stimulation with NRG1 and NMDA on Ca^2+ ^signalling. When NRG1 and NMDA were applied together, percentages of responsive cells and peak values were not significantly different from those obtained with NRG1 alone (99% of 218 cells; ΔR = 0.67 ± 0.03; Figure 5D). Plateau values, too, were not significantly different between the two conditions: NRG1, ΔR = 0.20 ± 0.007, n = 227; NRG1+NMDA, ΔR = 0.18 ± 0.008, n = 218.

Notably, time to peaks were significantly shorter as compared with those observed with separate stimulation with either NRG1 or NMDA (Additional file 5: Figure S5); this finding could imply that NMDA exerts some form of sensitization on the pathway dependent on ErbB4.

When NMDA was removed from the extracellular medium after the responses had already reached the plateau phase, a limited decay in the plateau could be observed (Figure 5B).

Finally, when NRG1 was applied during the slow response to NMDA (Figure 5C), a biphasic increase in [Ca^2+^]i was observed, with a peak amplitude not significantly different (ΔR = 0,603 ± 0,028; n = 42) from the responses to NRG1 alone.

## Discussion

Neuronal migration is a tightly regulated process in which the orchestrated activity of multiple molecules combines to bring about the appropriate allocation of post-mitotic nerve cells. Previous studies have shown that the NRG1/ErbB4 system is involved in several types of neuronal migration. On the other hand, intracellular calcium fluctuations, in many cases regulated by influx through Ca^2+ ^permeable membrane channels, play central roles in neuronal motility [32,34]; however, little is known about the involvement of calcium signals in NRG1-induced neuronal migration. In this work, we analyzed the calcium dependence of the migratory-promoting activity of the NRG1-ErbB4 system in immortalized neural progenitors (ST14A cells), and the interaction between this pathway and the one dependent on the NMDA-type glutamate receptor, a calcium permeable channel. Using ST14A cells exogenously expressing ErbB4, we demonstrate that the two systems synergistically act in stimulating progenitor cell migration in a Ca^2+^-dependent manner.

ST14A cells were chosen because they endogenously express all members of the ErbB family (ErbB1, ErbB2, and ErbB3) except ErbB4. We were able to compare migratory activity in cells lacking and expressing ErbB4 by using wild type ST14A cells and clones permanently transfected with specific ErbB4 isoforms [6]. Our RT-PCR studies show that ErbB4-transfected cells, similarly to wild type ST14A cells, express a restricted number of NMDAR subunits: NR1.4a, NR2C, NR2D and NR3B. Data from the literature indicate that the NR1 subunit is essential for NMDAR trafficking and insertion into the plasma membrane; different combinations of the NR2 and NR3 subunit families generate NMDA receptors differing in their functional characteristics, and cell-type and sub-cellular localization [31]. The two NR2 subunits expressed by ST14A cells (NR2C and NR2D) are prevalently extra-synaptic and are characterized by low-conductance openings and low sensitivity to Mg^2+ ^block [35-37]. On the other hand, NR3 subunits act like a dominant negative, reducing the amplitude of the response and its desensitization, and decreasing the Ca^2+ ^permeability of the NMDAR channel [38]. Since usually NMDARs are composed of at least one NR1 subunit and variable combinations of NR2/NR3 subunits, the resulting NMDARs would generate smaller, long lasting currents, that are less Mg^2+^-sensitive and Ca^2+^-permeable than those of NR2A-2B containing NMDARs.

We did not find a statistically significant effect of NMDA on basal migration of either wild type or ErbB4-transfected ST14A cells; this finding could be ascribed either to a low number of NMDA receptors on the ST14A cell membrane, or to their reduced Ca^2+ ^permeability, yielding a Ca^2+ ^influx not sufficient to elicit a migratory response by itself.

ErbB4 expression is necessary for NRG1-induced migration in ST14A progenitors. The mechanism has been partly characterized in our laboratory in previous studies [6]: ErbB4 dimerization and tyrosine kinase trans-phosphorylation is followed by intracellular signalling cascades involving PI3K activation. When NMDA was added to NRG1 in migration assays, we found a significant enhancement of migration compared to NRG1 alone (NRG1 alone: 5 fold, NRG1 + NMDA: 8 fold increase in migration rate). This strengthening of NRG1-stimulated migration is suggestive of a cooperation between NMDA and ErbB4 receptors in inducing and/or maintaining a migratory behaviour in ST14A cells. The NMDA-mediated increase in NRG1-stimulated migration could be prevented by the addition of a NR2C/D subunit-specific antagonist (UBP141), strongly suggesting that the NMDARs cooperating with ErbB4 during ST14A cells migration contain NR2C/D subunits.

The role of increases of the cytosolic calcium concentration in controlling cell motility has been reported in various cell types ranging from fibroblasts to immature neurons [39,40]. Neuronal precursors and post-migratory neurons in the fetal cerebrum and the early postnatal cerebellum exhibit spontaneous Ca^2+ ^transients [41,42], suggesting that the role of Ca^2+ ^transients in development may be constantly remodelled by internal programs and extracellular cues. Ca^2+ ^transients may affect the recycling of cell-adhesion receptors and induce the rearrangement of cytoskeletal components, which are essential for cell movement. The spontaneous Ca^2+ ^transients in migrating granule cells are mediated by NMDA receptors and by N-type Ca^2+ ^VDCCs [10,34]. Other agonists that can trigger an elevation in [Ca^2+^]_i _involved in motility control are the neurotransmitter GABA, that exerts an excitatory action in immature cells due to the high intracellular Cl^- ^concentration, the neuropeptide somatostatin that influences Ca^2+ ^fluctuations in a stage-dependent manner, and neurotrophins, like Brain Derived Neurotrophic Factor [13,43-45]. The ErbB4 receptor, like other members of the ErbB family [46,47], upon ligand activation could elicit calcium release from intracellular stores and calcium influx. However, there are no data available for this mechanism in neuronal cells, apart form a report [48] indicating that NRG1 can modulate glutamate-induced [Ca^2+^]_i _increase. Therefore, we checked if the synergism of NMDAR and NRG1/ErbB4 systems in enhancing ST14A migration could be due to a modulation of Ca^2+ ^signalling.

In migration experiments carried out in the presence of the intracellular Ca^2+ ^chelator BAPTA-AM we found a highly significant reduction both in NRG1 and in NMDA+NRG1 stimulated migration, with the latter reduced to the same level seen with NRG1 alone. This result strongly supports the hypothesis that both NRG1 induced migration in ST14A cells and the synergistic effect of NMDAR/ErbB4 activation involve intracellular Ca^2+ ^changes. For NRG1-induced migration, values in the presence of BAPTA-AM were still significantly higher than controls, indicating that NRG1/ErbB4-induced migration could involve also Ca^2+^-independent mechanisms.

In the attempt to understand the mechanisms underlying these calcium-dependent effects, we began to characterize the Ca^2+ ^signalling induced by NRG1 and NMDA by means of calcium imaging experiments. Our findings indicate that NRG1 stimulation of ErbB4-transfected ST14A cells induces a long lasting increase in [Ca^2+^]_i_, while no response could be recorded from mock transfected cells. To clarify the relative contribution and extent of calcium influx from the extracellular medium and of release from internal stores, NRG1 stimulation was repeated in a medium without Ca^2+ ^and containing the calcium chelator EGTA. Cells responded to NRG1 with an increase in free calcium, but the signal was significantly smaller and showed a transient time course. Intriguingly, after termination of NRG1 stimulation and subsequent reinstatement of Ca^2+ ^in the medium, the level of [Ca^2+^]_i _was strongly increased. This finding can be explained by the activation of a store-operated calcium entry (SOCE) driven by channels activated in response to depletion of the intracellular Ca^2+ ^stores (see e.g. 33). Accordingly, when the mechanism was activated by emptying the intracellular stores by means of thapsigargin, cells did not respond to a subsequent stimulation with NRG1.

Stimulation with NMDA led to an increase in free intracellular Ca^2+ ^that was significantly smaller than the one obtained with NRG1, could be observed in only 46% of cells tested and was completely abolished when the cells were preincubated either in a calcium-free solution or in the presence of the NR2C/D subunit-specific NMDAR antagonist UBP141. Significantly, these signals showed a very slow time course: more than 10 min were required to obtain a plateau value. This finding can be explained by a very low level of activation of NMDA channels at resting potentials, compatible with the proposed reduced Mg^2+ ^sensitivity of the subunits expressed in our cells; a limited activation of these channels could induce some depolarization, thus slowly recruiting additional channels. The slow time course of activation of this mechanism, and the relatively low global increase in calcium levels could not be sufficient to promote cell motility.

When both agonists were applied simultaneously, or when NRG1 was presented during the slow rising phase of the NMDA response, peak amplitudes were not significantly different from those obtained following stimulation with NRG1 alone; however, when NMDA was removed during the plateau phase of the response, that can be ascribed to calcium influx, a slight reduction in [Ca^2+^]_i _could be detected. Taken together, these observations can explain the different roles of the two agonists in promoting migration of ST14A cells and their calcium dependence: the slow and small increases in [Ca^2+^]_i _elicited by NMDAR activation are not sufficient to promote a significant amount of neuronal migration, while the faster, stronger and sustained signals induced by the NRG1/ErbB4 pathway are sufficient; however, when NMDA is presented together with NRG1, the small additional amount of calcium entering from the extracellular medium can exert a synergistic effect, possibly by inducing calcium signals localized to cellular subdomains (e.g. submembrane regions) that could contribute to the enhancement of cell motility.

NRG1 is one among several candidate genes linked to a relevant behavioural disease, schizophrenia. There are many hypotheses on the pathogenesis of this disease: some studies suggest that young-adult onset of schizophrenia may originate from disarranged circuitry caused by an altered migration of neuronal precursors during pre- and post-natal brain development [49,50]. According to post-mortem studies, in the brain of schizophrenic patients there is an altered NRG1/ErbB4 signalling and an hypo-functional glutamatergic system. Therefore, the understanding of NMDAR-NRG1/ErbB4 interactions during neural precursor migration may contribute to reveal the molecular and cellular basis of schizophrenia and of other neurodevelopmental disorders.

## Conclusions

In summary, we present in this paper the first experimental evidence that NRG1, by activating the ErbB4 receptor, can induce long lasting increases in intracellular calcium in a line of striatal neural progenitors, and that these signals are involved in the migratory behaviour of these cells. The mechanism responsible for these signals appears to be mainly dependent on the activation of a store operated calcium influx. Moreover, we suggest a role for NMDA receptors in modulating NRG1/ErbB4-induced migration. This interaction is calcium-dependent, even if the mechanism responsible for this cooperative effect still needs to be elucidated.

## Methods

### Reagents

All chemicals and reagents were purchased from Sigma (St. Louis, MO), unless otherwise stated. The EGF-like domain of mouse NRG1β1 (called NRG1 throughout the article) was produced in our laboratory as a His-tag fusion protein in *Escherichia coli *[51].

PPDA ((2R*,3S*)-1-(Phenanthrenyl-2-carbonyl)piperazine-2,3-dicarboxylic acid cis), UBP141 ((2R*,3S*)-1-(Phenanthrenyl-3-carbonyl)piperazine-2,3-dicarboxylic acid)), (+)-MK801 ((5S,10R)-(+)-5-Methyl-10,11-dihydro-5H-dibenzo[a, d]cyclohepten-5,10-imine maleate) and D-AP5 (D-(-)-2-Amino-5-phosphonopentanoic acid) were all purchased from Ascent Scientific (Bristol, UK). BAPTA-AM (1,2-Bis(2-aminophenoxy)ethane-N, N, N', N'-tetraacetic acid tetrakis acetoxymethyl ester) was purchased from Calbiochem. FURA-2-AM (Fura-2-acetoxymethyl ester) was from Molecular Probes Inc. All reagents were resuspended and stored following the manufacturer's instructions.

### Cell Culture

The ST14A cell line (kindly provided by prof. Elena Cattaneo), was derived from primary cells dissociated from rat striatal primordia at embryonic day 14 and conditionally immortalized by retroviral transduction of the temperature-sensitive variant of the SV40 large T antigen, as previously described [23]. Cells were cultured on 10 cm petri-dishes (BD Biosciences) in Dulbecco's modified Eagle's medium (DMEM) supplemented with 100 units/ml penicillin, 0.1 mg/ml streptomycin, 1 mM sodium pyruvate, 2 mM L-glutamine, and 10% or 2% fetal bovine serum (FBS, GIBCO^®^, Carlsbad, CA, USA) decomplemented at 56°C for 30 minutes. In this work we used an ST14A clone stably expressing the JMa/cyt2 ErbB4 isoform (clone A2.1). ErbB4-stable transfectants were previously obtained [6] subcloning the cDNA corresponding to this ErbB4 isoform into the pIRESpuro2 vector (Clontech) and stably transfecting wild type ST14A cells as previously described. ErbB4-transfected cells were grown in medium containing 5 μg/ml puromycin. Cells were grown as monolayers at the permissive temperature of 33°C in a 5% CO_2_, 95% air atmosphere saturated with H_2_O. Cells were allowed to grow to near confluence, and adherent cells were harvested by the trypsin/EDTA method.

### Dose-response assays

Preliminary drug dose-response assays were performed to identify the correct drug concentration to be used in migration experiments. NMDA dose-response treatment was carried out by plating 10^5 ^cells in triplicate on 24-well dishes, and treating cells for 18 hrs at 33°C with scalar concentrations of NMDA (1, 2, 4, 10 μM) in 2% FBS DMEM. For BAPTA-AM treatments, cells cultured in 2% FBS were incubated 20 min with BAPTA-AM (5, 10, 15 μM), then the medium was changed and the cells were incubated for 4 hrs at 33°C to avoid detachment and to allow complete de-esterification of the internalized BAPTA-AM; cells were then trypsinized and plated on 6-well dishes at the density of 5 × 10^4 ^cells/well and incubated at 33°C for 18 and 24 hrs. At the end of treatments, cells were washed with PBS with calcium, fixed in 2% glutaraldehyde for 20 min at RT, washed in water, stained with 0.1% crystal violet, 20% methanol for 20 min at RT, washed in water and then analyzed.

Dose-response assay for NMDAR antagonists was carried out plating 5 × 10^3 ^cells in 8 wells per condition on 96-multiwells. Cells were allowed to attach for 24 hrs in 10% FBS DMEM, then the medium was replaced with 2% FBS containing three concentration for each antagonist: PPDA (0.2-0.5-1 μM), UBP141 (2-5-10 μM), MK-801 (5-10-20 μM), D-AP5 (10-50-100 μM). After 18 hrs, cells were fixed in 2% glutaraldehyde for 20 min at room temperature and then stained with 0.1% crystal violet, 20% methanol solution for 20 min, washed in water, and air-dried. Bound dye was solubilized by addition of 10% acetic acid (100 μl/well) and 5 min of shaking at room temperature. The absorbance of dye extracts was measured directly in plates using a Microplate Reader (Bio-Rad) at a wavelength of 590 nm.

### Transwell Assays

Transwell assays were carried out using cell culture inserts (Cat. #353097, BD Biosciences) as described in [6], with minor modifications. 7 × 10^4 ^cells resuspended in 200 μl 2%FBS DMEM were seeded in the upper chamber of the cell culture insert. The lower chambers contained 0.8 ml 2%FBS DMEM as the control condition, or medium added with NRG1 and/or NMDA and/or NMDA antagonists (5 nM NRG1, 8 μM NMDA, 5 μM UBP141, 0.5 μM PPDA, 10 μM MK-801, 30 μM D-AP5). In migration experiments performed with the calcium-chelator BAPTA-AM, cells were pretreated 20 min in the culture plate with 15 μM BAPTA-AM in 2%FBS-DMEM, then cultured 60 min in 2%FBS DMEM, detached and counted for the transwell assay (control cells were incubated 80 minutes in 2%FBS-DMEM). In migration experiments performed with NMDA receptor antagonists, (UBP141, PPDA, MK-801, D-AP5) cells resuspended in 2%FBS-DMEM (3, 5 × 10^4 ^cells/ml) were preincubated 20 minutes with antagonists, then 200 μl (7 × 10^4^) cells were seeded in each upper chamber. Cells were allowed to migrate 18 hrs at 33°C. At the end of the incubation time, inserts were washed twice in PBS, and the top side of the membrane was wiped clean with cotton tipped applicators to eliminate un-migrated cells. Migrated cells present on the lower side of the filter were fixed in 2% glutaraldehyde for 20 minutes at room temperature and then stained with 0,1% crystal violet, 20% methanol solution for 20 minutes, washed in water, air-dried and then photographed (four 8-bit images per well, 4× magnification) using an inverted microscope Olympus IX50 equipped with a Cool SNAP-Pro CCD camera. All conditions were performed in triplicate. Four images were analyzed for each transwell using the ImageJ software and the amount of migrated cells was evaluated as the total area of migration (in pixel^2^) and then expressed as percentage of the total number of migrated cells for every single experiment, in order to normalize the variability between different experiments. Cells were discriminated by the pores of the Transwell membrane by applying a threshold of 300 pixel^2^. Statistical analysis was performed using SPSS software on technical triplicates of three different experiments. The significance of effects of treatment was assessed using the one or two-way ANOVA followed by Tukey's multiple comparisons with p < 0.05 considered significant.

### RNA extraction and Reverse Transcription (RT)-PCR

Total RNA extraction was performed using TRIZOL^® ^Reagent (Invitrogen Life-Technologies) using a modified protocol compared to the manufacturer's instruction. Briefly, following washing in PBS, 800 μl TRIZOL were added to the dishes, cells were collected with a cell scraper and then transferred to a microcentrifuge tube on ice. Samples were homogenized by several passages through a 22G needle connected to a 1 ml syringe. Following the addition of chloroform and centrifugation, the aqueous phase was transferred to a fresh tube and RNA precipitated by isopropyl alcohol. The RNA pellet was resuspended in 20 μl sterile water and treated with DNAse (Promega) according to manufactory instructions. RNA was re-extracted in TRIZOL and quantified by measuring absorbance at 260/280 nm; all samples were diluted to the final concentration of 0.25 μg/μl.

Total RNA was reverse-transcribed into cDNA in a reaction volume of 20 μl, containing 10 U M-MuLV Reverse Transcriptase (Fermentas), 2.5 μM random hexamers (Fermentas), 50 mM Tris-HCl (pH 8.3 at 25°C), 75 mM KCl, 3 mM MgCl_2_, 10 mM DTT, 1 U/μl RiboLock™ RNase Inhibitor (Fermentas), 1 mM deoxynucleotide triphosphates (dNTPs, Amersham-Biosciences). RNA samples were denaturated 5 min at 80°C, then incubated in the reaction mixture for 5 to 10 min at 25°C followed by 60 min at 42°C and 5 min at 95°C. cDNA products were diluted 1:3 in sterile water and stored at -20°C.

PCR reactions were carried out for 30 (G3PDH) or 35 cycles (all other genes) in a total volume of 30 μl including 3 μl cDNA, 0.5 μM of each primer pair, 1× standard reaction buffer (10 mM Tris-HCl pH 8.3, 50 mM KCl, 1.1 mM MgCl_2_, 0.01% jelly), 200 μM dNTPs (Amersham) and 0.025 U/μl of REDTaq DNA polymerase. Primer sequences, size of amplification products and annealing temperatures are listed in Table 1. G3PDH amplification was used to monitor the quality and quantity of RNA that had been reverse-transcribed into cDNA. Cycling parameters were set for each primer pair in order to obtain specific products in the exponential amplification phase of the PCR reaction. Negative controls consisted in removal of reverse transcriptase (RT-) from RT reaction and in replacement of cDNA with sterile water (C-) in PCR reactions. Amplification products were separated by agarose gel electrophoresis and DNA bands were visualized with ethidium bromide staining and gel images were digitalized by Gel Doc (Bio-Rad).

**Table 1:** PCR amplification primers

Gene	Primer Sequence	Size (bp)	T(°C)
G3PdH	5'-TGGCATTGTGGAAGGGCTCATGAC-3'	188	60
	5'-ATGCCAGTGAGCTTCCCGTTCAGC-3'		
NR1 N-ter	5'-AGTCCAGCGTCTGGTTTGAGATGA-3'	235(a)/298(b)	66
	5'-CTCGCTTGCAGAAAGGATGATGAC-3'		
NR2A	5'-TTCATCTGGGAGCACCTCTT-3'	402	60
	5'-GGCCACAAATGTTTGGAGTT-3'		
NR2B	5'-GGCATTGCTATCCAAAAGGA-3'	449	60
	5'-GACAGGTTGGCCATGTTCTT-3'		
NR2C	5'-GCACACCCACATGGTCAAGTTC-3'	327	60
	5'-CTGCTCATCACCTCATTCTTCT-3'		
NR2D	5'-GCAATACCCACCTCTGAAGTTT-3'	350	60
	5'-CCAGCATCTCAATCTCATCGTC-3'		
NR3A	5'-GGTTCCCTGCGGAAAGAGAAGC-3'	310/370	60
	5'-TACCATGAGCTGCTGGGGTCCC-3'		
NR3B	5'-GGTTGGGGACAAAACCTTTGAGG-3'	287	60
	5'-CGGTGAGCAGCTTGCAGTCC-3'		
NR1 C-ter	5'-ATGCCCCTGCCACCCTCACTTTTG-3'	381(NR1.1)/270(NR1.2)	70
	5'-GCAGCTGGCCCTCCTCCCTCTCA-3'		
NR1 C-ter	5'-TACAAGCGACACAAGGATGC-3'	241(NR1.3)/132(NR1.4)	60
	5'-CGAGGGATCTGAGAGGTTGA-3'		

### Calcium imaging

#### Loading of Ca^2+ ^Indicator Dye

The [Ca^2+^]_i _in ST14A cells was monitored using the Ca^2+ ^indicator dye FURA-2-AM. ST14A cells were plated on poly-L-lysine coated glass coverslips contained in 60 mm dishes at a density of 5*10^3 ^cells/cm^2 ^and left 3 days in 10% FBS, after which the medium was changed to 2% FBS DMEM about one hour before the experiment. Cells were loaded for 45 min at 37°C with 3 μM FURA-2-AM in 2% FBS DMEM and subsequently washed in Tyrode Standard Solution (Tyr-St: NaCl 154 mM, KCl 4 mM, CaCl_2 _2 mM, MgCl_2 _1 mM, HEPES 5 mM, glucose 5.5 mM, pH 7.34 with NaOH).

#### Calcium Measurements

After dye loading cells were transferred in a perfusion chamber connected to a peristaltic pump and mounted on an inverted microscope (Nikon TE-2000-S) equipped with 20× magnification, a Xenon lamp illumination system and a CCD camera. All experiments were performed at room temperature. A gravity microperfusion system, regulated by electrovalves, was employed to allow the switch from the standard Tyrode solution to the same medium containing drugs (5 nM NRG; 8 μM NMDA; 10 μM UBP141). Some experiments were performed in a Tyrode solution without calcium (Tyr-0-Ca^2+^, 157 mM NaCl, 4 mM KCl, 1mM MgCl_2_, 5 mM HEPES, 5.5 mM glucose, 0.5 mM EGTA, pH 7.34 with NaOH) to evaluate the contribution of calcium release from intracellular stores. Calcium measurements were performed exciting FURA-2-AM every 0.8 s alternatively at 340 nm and 380 nm, and emission was recorded at 510 nm. For each experiment ROIs (Region of Interest) corresponding to the cell bodies were chosen. The obtained fluorescence values were plotted against time to generate graphics of the intracellular Ca^2+ ^trend in response to drug administration. Images were visualized on a computer with the dedicated acquisition software Metafluor (Universal Imaging Corporation, PA). Data were analyzed with the IGOR Pro software (6.03a version). Each experiment was performed three times on different cell cultures, peak values were calculated subtracting the baseline value for each trace from the maximum value reached during drug treatment. The statistical analysis was carried out using SPSS software (descriptive statistics, ANOVA and non parametric test) and data are represented as mean ± standard error or as percentages.

## Authors' contributions

RT-PCR experiments were designed by PB and GP, and carried out by GP. Recombinant NRG1 was produced by FF. ErbB4 transfected clones were produced and characterized by GG. Transwell assays were designed by PB and GG, carried out and analyzed by GP and VL. Long-term experiments with BAPTA-AM were designed by SF and DL, performed and analyzed by GP and VL. Calcium imaging experiments were designed and performed by PZ and GP, and analyzed by PZ. General study coordination was carried out by PB, manuscript drafting by PB and DL. Critical revision of the manuscript was performed by IP, GG and PZ. All authors read and approved the final manuscript.

## Supplementary Material

Additional File 1 **Figure S1 - Different NMDAR antagonists are able to reduce the (NRG1+NMDA)-induced migration**. ErbB4-transfected ST14A cells were treated with 8 μM NMDA + 5 nM NRG1 for 18 hrs (control condition) in the presence of various NMDA receptor antagonists: PPDA (0.5 μM), UBP141 (5 μM), MK801 (10 μM) and D-AP5 (30 μM). Migration was calculated as percentage of total migrated cells as described in Material and Methods. Data are presented as means + SD (n = 3). Statistical analysis was performed by one-way ANOVA followed by Tuckey's test, *** = p < 0.001, ** = p < 0.01, * = p < 0.05. Click here for file

Additional file 2 **Figure S2 - The NR2C/D subunit-selective antagonist UBP141 is able to inhibit the NMDA effect on NRG1-induced migration**. ErbB4-transfected cells were treated for 18 hrs with different combinations of 8 μM NMDA, 5 nM NRG1, 5 μM UBP141 and 15 μM BAPTA-AM. Notice that UBP141 has no effect *per se *on ST14A cell migration and that the presence of BAPTA completely masks the inhibitory effect of UBP141 on (NRG1+NMDA)-induced migration. Migration was calculated as percentage of total migrated cells as described in Material and Methods. Biological triplicate experiments were carried out in technical triplicate. Data are presented as means + SD. Statistical analysis was performed by one-way ANOVA followed by Tuckey's post hoc test, significant differences vs control are indicated as * = p < 0.05; ** = p < 0.01; *** = p < 0.001, while significant effects between treatments are indicated as # p < 0.05; ## = p < 0.01; ### = p < 0.001. Click here for file

Additional file 3 **Figure S3 - The NMDAR antagonist UBP141 suppresses NMDA-induced calcium signals**. In the presence of 10 μM UBP141, a NR2C/D subunit-specific NMDAR antagonist, stimulation with 8 μM NMDA failed to induce a response in 100% of 57 cells. A representative trace is shown. Click here for file

Additional file 4 **Figure S4 - Responses to NMDA are abolished in the absence of extracellular calcium**. When cells were preincubated in an extracellular solution containing 0 Ca^2+ ^and 0.5 mM EGTA, no response to 8 μM NMDA could be detected in 100% of 80 cells. The mean ± SE is shown. Click here for file

Additional file 5 **Figure S5 - Times to peak of calcium increases induced by the different agonists**. Times to peak of the calcium responses to NRG1 (n = 224), NMDA (n = 126) and to the combined presence of the two agonists (n = 215). Values were respectively 290.5 ± 14.1; 592.4 ± 13.2 and 179.6 ± 3.8. ++ p < 0.01 vs. NRG1; ** p < 0.01 vs. NMDA. Click here for file
