# Supplementary figures and images for: Neuregulin1/ErbB4-induced migration in ST14A striatal progenitors: calcium-dependent mechanisms and modulation by NMDA receptor activation
Source: BMC Neurosci. 2011 Oct 12;12:103. doi: 10.1186/1471-2202-12-103 (PMC3209446; doi:10.1186/1471-2202-12-103)

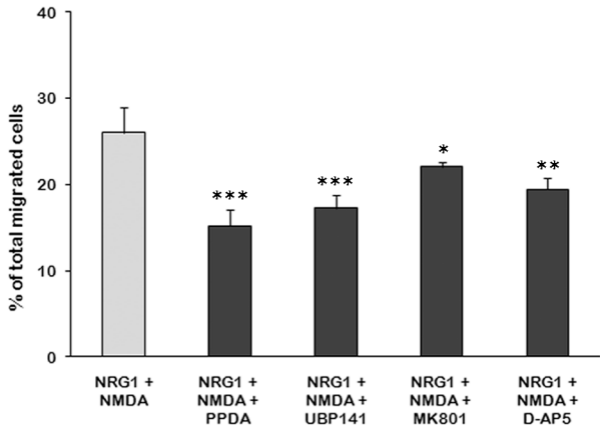

Supplement: Additional File 1 — Figure S1 - Different NMDAR antagonists are able to reduce the (NRG1+NMDA)-induced migration. ErbB4-transfected ST14A cells were treated with 8 μM NMDA + 5 nM NRG1 for 18 hrs (control condition) in the presence of various NMDA receptor antagonists: PPDA (0.5 μM), UBP141 (5 μM), MK801 (10 μM) and D-AP5 (30 μM). Migration was calculated as percentage of total migrated cells as described in Material and Methods. Data are presented as means + SD (n = 3). Statistical analysis was performed by one-way ANOVA followed by Tuckey's test, *** = p < 0.001, ** = p < 0.01, * = p < 0.05. [file 1471-2202-12-103-S1.PDF]

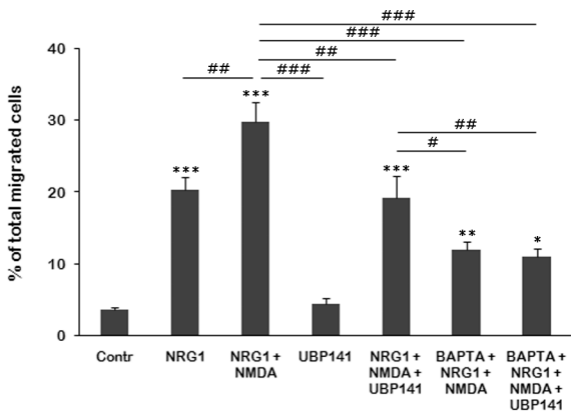

Supplement: Additional file 2 — Figure S2 - The NR2C/D subunit-selective antagonist UBP141 is able to inhibit the NMDA effect on NRG1-induced migration. ErbB4-transfected cells were treated for 18 hrs with different combinations of 8 μM NMDA, 5 nM NRG1, 5 μM UBP141 and 15 μM BAPTA-AM. Notice that UBP141 has no effect per se on ST14A cell migration and that the presence of BAPTA completely masks the inhibitory effect of UBP141 on (NRG1+NMDA)-induced migration. Migration was calculated as percentage of total migrated cells as described in Material and Methods. Biological triplicate experiments were carried out in technical triplicate. Data are presented as means + SD. Statistical analysis was performed by one-way ANOVA followed by Tuckey's post hoc test, significant differences vs control are indicated as * = p < 0.05; ** = p < 0.01; *** = p < 0.001, while significant effects between treatments are indicated as # p < 0.05; ## = p < 0.01; ### = p < 0.001. [file 1471-2202-12-103-S2.PDF]

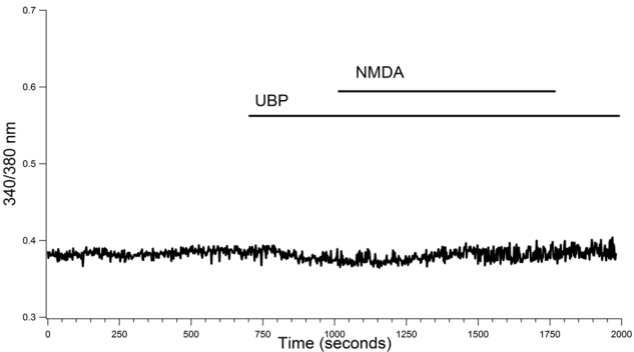

Supplement: Additional file 3 — Figure S3 - The NMDAR antagonist UBP141 suppresses NMDA-induced calcium signals. In the presence of 10 μM UBP141, a NR2C/D subunit-specific NMDAR antagonist, stimulation with 8 μM NMDA failed to induce a response in 100% of 57 cells. A representative trace is shown. [file 1471-2202-12-103-S3.PDF]

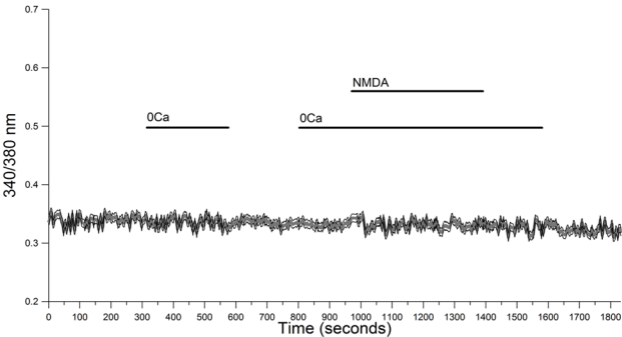

Supplement: Additional file 4 — Figure S4 - Responses to NMDA are abolished in the absence of extracellular calcium. When cells were preincubated in an extracellular solution containing 0 Ca2+ and 0.5 mM EGTA, no response to 8 μM NMDA could be detected in 100% of 80 cells. The mean ± SE is shown. [file 1471-2202-12-103-S4.PDF]

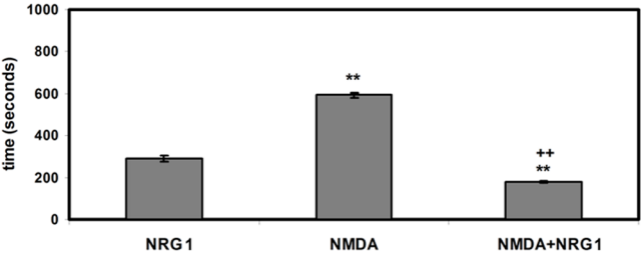

Supplement: Additional file 5 — Figure S5 - Times to peak of calcium increases induced by the different agonists. Times to peak of the calcium responses to NRG1 (n = 224), NMDA (n = 126) and to the combined presence of the two agonists (n = 215). Values were respectively 290.5 ± 14.1; 592.4 ± 13.2 and 179.6 ± 3.8. ++ p < 0.01 vs. NRG1; ** p < 0.01 vs. NMDA. [file 1471-2202-12-103-S5.PDF]
